# Uncovering phylogenetic relationships and genetic diversity of water dropwort using phenotypic traits and SNP markers

**DOI:** 10.1371/journal.pone.0249825

**Published:** 2021-07-06

**Authors:** Qun Ji, Honglian Zhu, Xinfang Huang, Kai Zhou, Zhengwei Liu, Yalin Sun, Zhixin Wang, Weidong Ke

**Affiliations:** Institute of Vegetables, Wuhan Academy of Agricultural Sciences, Wuhan, Hubei, China; National Cheng Kung University, TAIWAN

## Abstract

The water dropworts *Oenanthe linearis* Wall. ex DC. and *O*. *javanica* (Blume) DC. are aquatic perennial herbs that have been used in China as vegetables and traditional medicines. However, their phylogenetic relationships and genetic diversity are poorly understood. Here, we presented the phenotypic traits and genome-wide DNA marker-based analysis of 158 water dropwort accessions representing both species. The analysis revealed that *Oenanthe linearis* was readily segregated into linear-leaf and deep-cleft leaf water dropworts according to their leaf shapes at flowering. *Oenanthe javanica* was classified by clustering analysis into two clusters based mainly on the morphological characteristics of their ultimate segments (leaflets). A set of 11 493 high-quality single-nucleotide polymorphisms was identified and used to construct a phylogenetic tree. There was strong discrimination between *O*. *linearis* and *O*. *javanica*, which was consistent with their phenotype diversification. The population structure and phylogenetic tree analyses suggested that the *O*. *linearis* accessions formed two major groups, corresponding to the linear-leaf and deep-cleft leaf types. The most obvious phenotypic differences between them were fully expressed at the reproductive growth stage. A single-nucleotide polymorphism-based analysis revealed that the *O*. *javanica* accessions could be categorized into groups I andII. However, this finding did not entirely align with the clusters revealed by morphological classification. Landraces were clustered into one group along with the remaining wild accessions. Hence, water dropwort domestication was short in duration. The level of genetic diversity for *O*. *linearis* (π = 0.1902) was slightly lower than that which was estimated for *O*. *javanica* (π = 0.2174). There was a low level of genetic differentiation between *O*. *linearis* and *O*. *javanica* (Fst = 0.0471). The mean genetic diversity among accessions ranged from 0.1818 for the linear-leaf types to 0.2318 for the groupII accessions. The phenotypic traits and the single-nucleotide polymorphism markers identified here lay empirical foundation for future genomic studies on water dropwort.

## Introduction

The genus *Oenanthe* L. (Apiaceae) contains ~30 species that are widely distributed throughout Asia, Africa, Europe and North America [1]. *Oenanthe* is an aquatic perennial herb with widely hydrochorous dispersal [1]. *Oenanthe javanica* (Blume) DC. is consumed as a vegetable in China [2, 3], and contains moderate amounts of macronutrients, minerals, polyphenols, and carotenoids, and large quantities of lutein [4]. *Oenanthe javanica* has been used in traditional Chinese medicine. It contains isorhamnetin [5], hyperoside [6] and persicarin [7], all of which have pharmacological activities.

Nine species and one variety of *Oenanthe* native to China are recorded in the Chinese version of the “Flora of China” [8]. In the revised English translation of the “Flora of China”, only five *Oenanthe* species and six subspecies are recorded [1]. In recent years, progress has been made in the elucidation of phylogenetic relationships within subfamily Apiodeae [9, 10], tribe Oenantheae [11, 12] and genus *Oenanthe* [13] of the Apiaceae family via polymerase chain reaction (PCR) and direct DNA sequencing. Based on complete chloroplast genome sequences, the phylogenetic relationships among *O*. *javanica* and 34 related species from 29 Apiaceae genera were analyzed [14]. The taxonomic status of *Oenanthe* spp. in Apiaceae was investigated extensively in the aforementioned studies. However, only a few investigations focused on the phylogentic relationships among *Oenanthe* species [13] or the levels of genetic variation within this taxa [13, 15, 16]. A previous attempt to classify of 133 *Oenanthe linearis* Wall. ex DC. and *O*. *javanica* accessions relied on morphological traits. It categorized *Oenanthe linearis* accessions into linear-leaf and deep-cleft leaf types and divided *O*. *javanica* accessions into small-leaf and big-leaf types [17].

Genomic tools such as molecular markers clarify the taxonomic status, phylogenetic relationships, genetic diversity, and population structure of plant genetic resources. This information could be applied towards genetic improvement. Only a few studies have implemented nuclear rDNA internal transcribed spacer sequences (nrDNA ITS) [13], enzyme electrophoresis [15], random amplified polymorphic DNA [16], or simple sequence repeats [18, 19] as molecular markers to study genetic diversity and variation in *Oenanthe* accessions. However, they only used a few markers and representative *Oenanthe* accessions within a limited geographical region. In contrast, single-nucleotide polymorphisms (SNPs) are far more powerful, abundant, and stable than traditional markers. Provided that a reference genome is available, SNPs can be generated by whole genome resequencing. However, they are not cost-effective [20]. For species with or without reference genomes, SNPs are identified via genome complexity-reduction protocols such as genotyping-by-sequencing [21], specific length amplified fragment sequencing [22] and restriction site-associated DNA sequencing (RAD-seq) [23]. These modalities are inexpensive and have high throughput. These and other high-throughput DNA sequencing technologies have been applied to many species in various types of genetic/genomic research, such as quantitative trait loci mapping [24], phylogenetic analyses [25], crop origin and domestication [26, 27], genome-wide association studies [28, 29], and functional markers development [30].

Several *Oenanthe* species may have been cultivated and used as vegetables in different regions of China, they were classified as *O*. *javanica* and categorized as pointed-leaf or round-leaf type by local farmers and researchers [2, 3]. In our field investigation, we identified two species of widely cultivated *Oenanthe* based on their morphological characteristics and established that the pointed-leaf and round-leaf types corresponded to *O*. *linearis* and *O*. *javanica*, respectively. It is already known that *O*. *linearis* and *O*. *javanica* differ in terms of leaf shapes. However, we wanted to know whether different phenotypes exist within these species. To preserve and sustainably use the *Oenanthe* germplasm, we collected *Oenanthe* accessions and maintained them at the Wuhan National Germplasm Repository for Aquatic Vegetables. We studied the phylogenetic relationships and genetic diversity of 158 *O*. *linearis* and *O*.*javania* accessions collected across a wide geographical area in China and its neighboring countries. To these ends, we developed an integrated workflow by combining the morphological traits and RAD-seq-generated SNPs to discover phenotypic and genome-wide variations in water dropwort. This information could lay empirical foundation for the collection, preservation, evaluation and utilization of these genetic resources.

## Materials and methods

### Plant materials and DNA extraction

**Fig 1:**
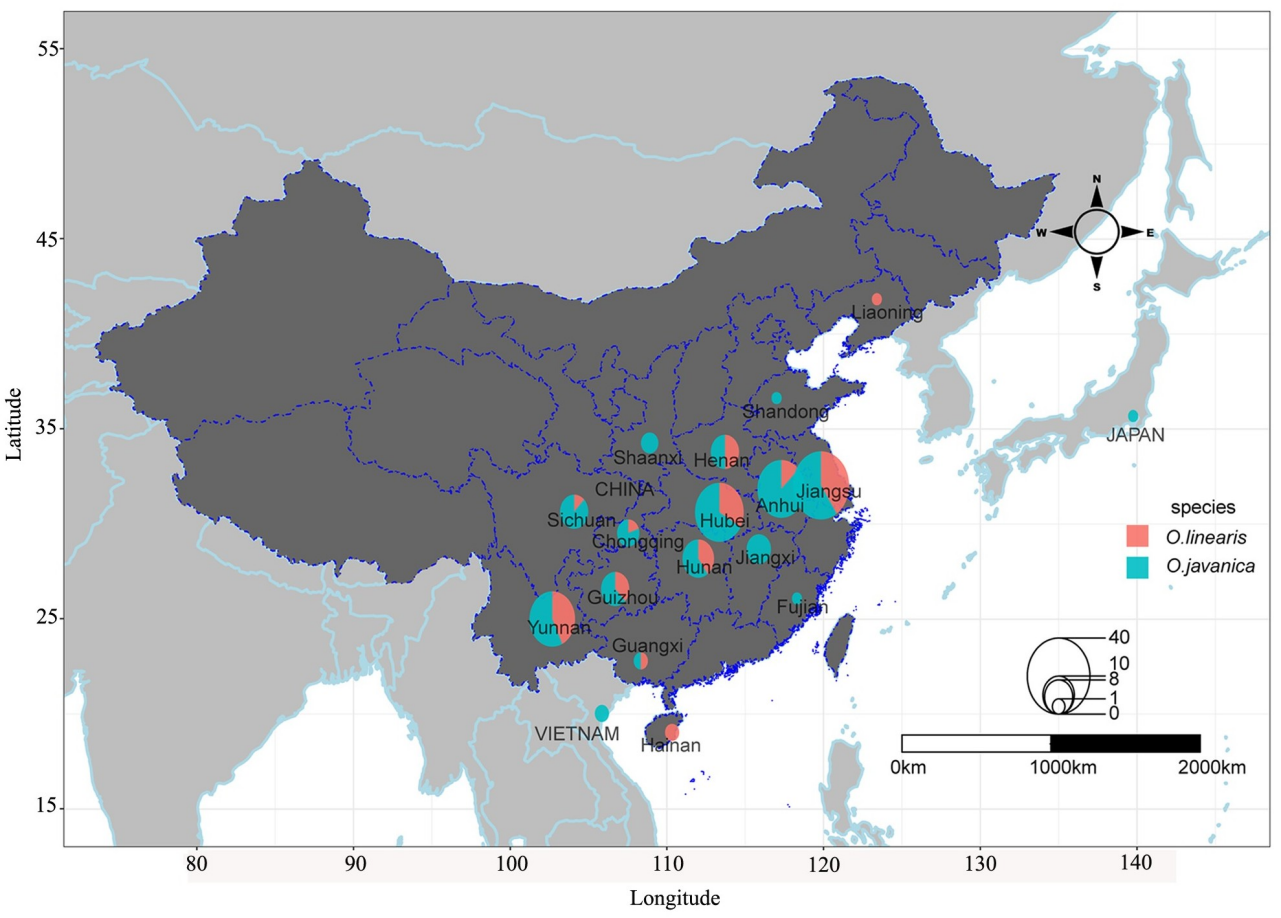
Geographic distribution of water dropwort accessions in China and its neighboring countries used in this study. Circle size is proportional to the number of accessions originating within a particular region. Different colors represent proportions of different species. Accessions of *O*. *linearis* and *O*. *javanica* mainly from eastern, middle-southern, and southwestern China, especially from Jiangsu, Anhui, Hubei, and Yunnan Provinces.

The plant materials included 50 *O*. *linearis* and 108 *O*. *javanica* accessions. Seven *O*. *linearis* and 18 *O*. *javanica* accessions were landraces, while the remaining 133 were wild. Twenty-three of the landraces were localized to the main distribution areas of China, whereas one was found in Japan and another was identified in Vietnam. A total of 132 wild water dropwort were collected from the natural distribution areas of China and one wild water dropwort was collected in Vietnam (Fig 1, S1 Table). Of the 158 accessions, 155 were from China including 32 from Jiangsu, 24 from Hubei, 23 from Anhui, 21 from Yunnan, 10 from Hunan, eight each from Henan, Guizhou and Sichuan, six from Jiangxi, five from Chongqing, three from Shaaxi, two each from Hainan and Guangxi, and one each from Fujian, Shandong, and Liaoning. Hence, the distribution areas in China were over-represented. All 158 accessions were sampled from the Wuhan National Germplasm Repository for Aquatic Vegetables.

Bulked young leaves from each accession were collected for genomic DNA extraction with a DP305 DNA extraction kit (Tiangen, Beijing, China) following the manufacturer′s instructions. DNA quality and concentration were evaluated by separation on a 1% agarose gel and reading in a NanoDrop-2000 spectrophotometer (Thermo Fisher Scientific, Waltham, MA, USA). The DNA concentrations were adjusted to 50 ng μl^-1^.

### Phenotypic traits

The 158 accessions were individually planted and vegetatively propagated on 6-m^2^ plots at the Wuhan National Germplasm Repository for Aquatic Vegetables. The key morphological and agronomical characteristics, plant propagation pathway (rhizome or stolon), flowering time, plant height, stem diameter, leaf shape, leaf state (even or uneven), leaf margin, and ultimate segment (leaflet) length and width were measured and recorded for six representative plants per plot per year during 2016–2018. Data processing including univariate ANOVA and cluster analysis were performed in SPSS v. 22 (IBM Crop., Armonk, NK, USA). SPSS TwoStep cluster analysis was applied because its algorithm creates clusters based on both categorical and continuous variables [31]. Clustering was analyzed using the log-likelihood algorithm based on the categorical variables leaf state and margin and the continuous variables flowering time, plant height, stem diameter, and ultimate segment length: width ratio.

### RAD sequencing, read clustering, and SNP exploitation

RAD library preparation, sample indexing, and pooling were conducted for all accessions according to Baird et al. [32]. A DNA digestion experiment was performed to test various restriction enzymes and established that *Ape*KI should be used to digest the DNA. The fragments were ligated to the Solexa P1 adapter containing a unique barcode for each DNA sample. The 300–700-bp adapter-ligated fragments were collected to ligate the P2 adapter. Only fragments with both P1 and P2 adapters could be enriched by PCR amplification. Amplified fragments ranging from 200–400 bp and 400–600 bp were selected for library construction. Paired-end sequencing was performed with an Illumina HiSeq2500 (Illumina Inc., San Diego, CA, USA). For QC, the ratio of sequencing nucleotides with quality value > Q30 (0.1% sequencing error) was determined, and the GC content was also assayed. High-quality reads were trimmed from the 3’ end to 150 bp. Trimmed reads with clear index information were clustered into RAD-tags via *ustacks* based on their sequence similarity [33] to generate candidate alleles for each locus. The RAD-tags were grouped by *cstacks* into clusters under the default SNP calling parameters. SNPs with minor allele frequency (MAF) < 0.05 and integrity < 0.8 across genotype datasets for all accessions were discarded.

### Phylogenetic relationship, population structure, and genetic diversity analyses

SNP genotype was used to calculate the genetic distances among the 158 accessions. Reliable and accurate phylogenetic tree was generated using the neighbor-joining (NJ) method in MEGA5 [34]. A bootstrap consensus tree was obtained using 1,000 replicates. Population structure was analyzed in ADMIXTURE based on the maximum-likelihood method [35]. Five independent runs were performed per *K* value with a 1–10 range constraint. Accessions were assigned to corresponding memberships with groups based on their maximum membership probabilities. Phylogenetic tree and population structure analyses were validated using principal component analysis (PCA), conducted in Cluster v. 3.0 [36]. The Stacks populations program [37] was applied to calculate nucleotide diversity (π) and pairwise differentiation levels (Fst). Nei’s genetic diversity index and Nei’s genetic distance were calculated in the R packages "snpReady" and " StAMPP ", respectively [38, 39].

SNPs discovered by RAD-seq and used in genetic diversity estimation might introduce error by misalignments sequences via paralogy. Random subsampling assesses the stability of phylogenetic analyses by selecting random locus subsamples and comparing their phylogenetic analyses [40]. Here, SNPs were sampled 10 times at random from the total dataset at a 1:10 frequency to build 10 different subsamples for population structure analyses and to test for consistency among various SNP subsample analyses.

## Results and discussion

### Phenotypic differences between *O*. *linearis* and *O*. *javanica*

*Oenanthe linearis* and *O*. *javanica* leaves tended to be pointed and round respectively, especially at flowering. The mean length:width ratios of ultimate segments were 2.27 and 1.65 for *O*. *linearis* and *O*. *javanica* respectively (S2 Table). The rhizomes of *O*. *linearis* arising from the axillary buds on the underground nodes and stolons of *O*. *javanica* arising from the axillary buds in the leaf axils could develop into offshoots. The flowering time of *O*. *linearis* was ~20 d earlier than that of *O*. *javanica*. The former started to flower in early May, whereas the latter began to flower in late May (S2 Table).

Fifty accessions were recorded as *O*.*sinensis* Dunn by the Wuhan National Germplasm Repository for Aquatic Vegetables according to the Chinese version of the “Flora of China” [8]. However, they were re-classified as *O*. *linearis* based on the revised English translation of the “Flora of China” wherein *O*.*sinensis* is synonymous with *O*. *linearis* [1].

### Phenotypic differences among *O*. *linearis* accessions

**Fig 2:**
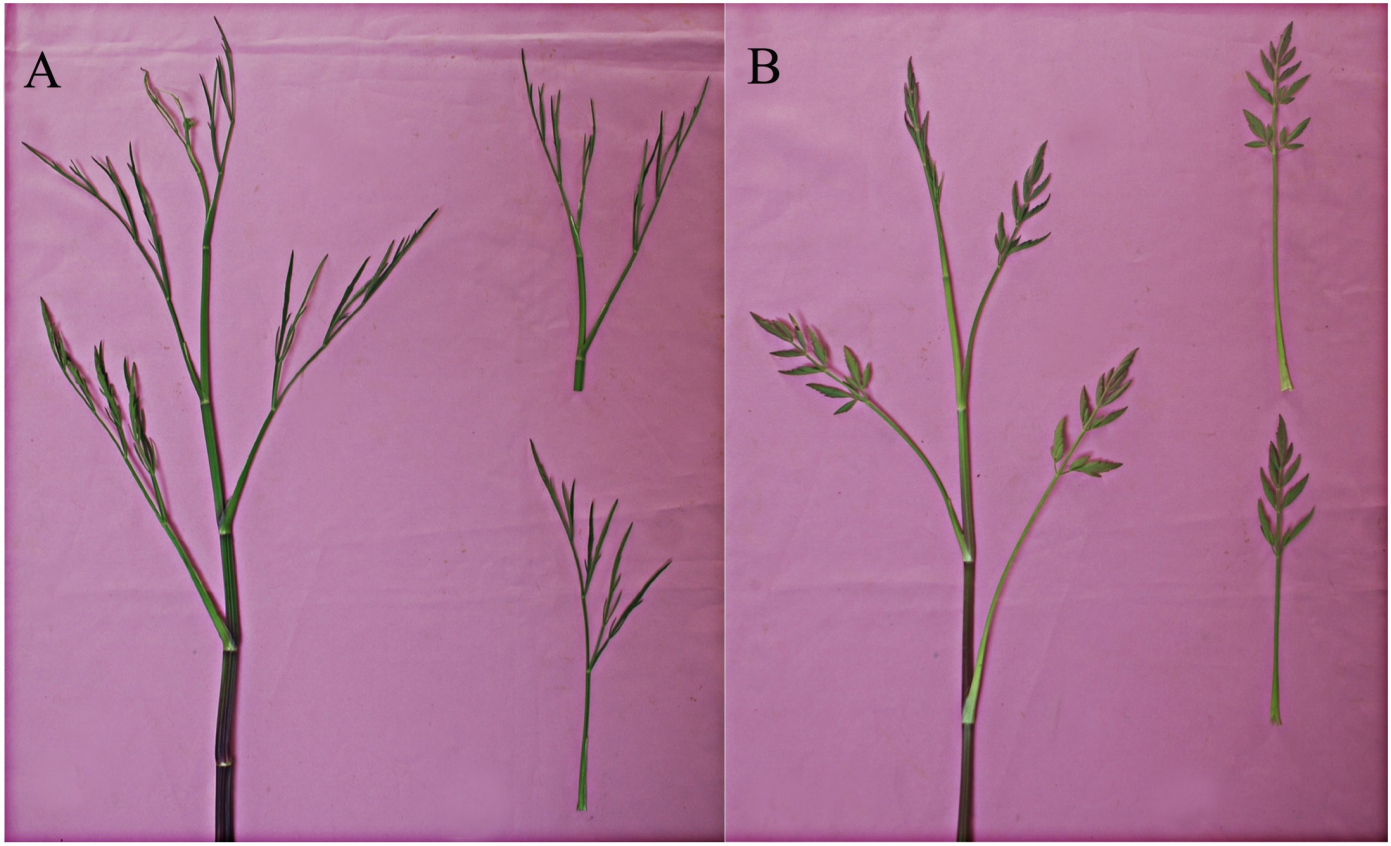
Morphology of the two types of *O*. *linearis*. (A) Linear-leaf type accession; (B) Deep-cleft leaf type accession.

Of the 50 aforementioned *O*. *linearis* accessions, 30 were LL and 20 were DCL types (Fig 2, S2 Table). There were no obvious morphological differences between LL and DCL during vegetative growth. However, as the plants entered reproductive growth, it was easier to distinguish LL from DCL according to their leaf shapes. As a rule, LL flowered ~15 d earlier than DCL (S2 Table).

The mean DCL plant height (65.71 cm) was greater than that of LL (61.70 cm), but these differences were not statistically significant (*P* = 0.296). There were also no significant differences between LL and DCL in terms of stem diameter (*P* = 0.107) (LL: 0.67 cm; DCL: 0.79 cm) (S2 Table).

Ye et al. [17] analyzed 35 *O*. *linearis* accessions and proposed that they could be classified into LL and DCL types based on their leaf shapes. In the present study, *O*. *linearis* was readily categorized into LL and DCL based on their leaf shapes and flowering time. Mean plant height and stem diameter were larger for the DCL than the LL types. Though these findings corroborate those of Ye et al., univariate ANOVA was not conducted in the prior work. Within *O*. *linearis*, five accessions were apparently intermediate between LL and DCL types. Most *O*. *linearis* accessions could be easily categorized as LL or DCL after bolting. The five aforementioned accessions were classified as DCL but identified as LL at full bloom. Thus, the *O*. *linearis* phenotype should be accurately identified at full bloom, as its leaf morphology is completely expressed by that time.

### Phenotypic differences among *O*. *javanica* accessions

**Fig 3:**
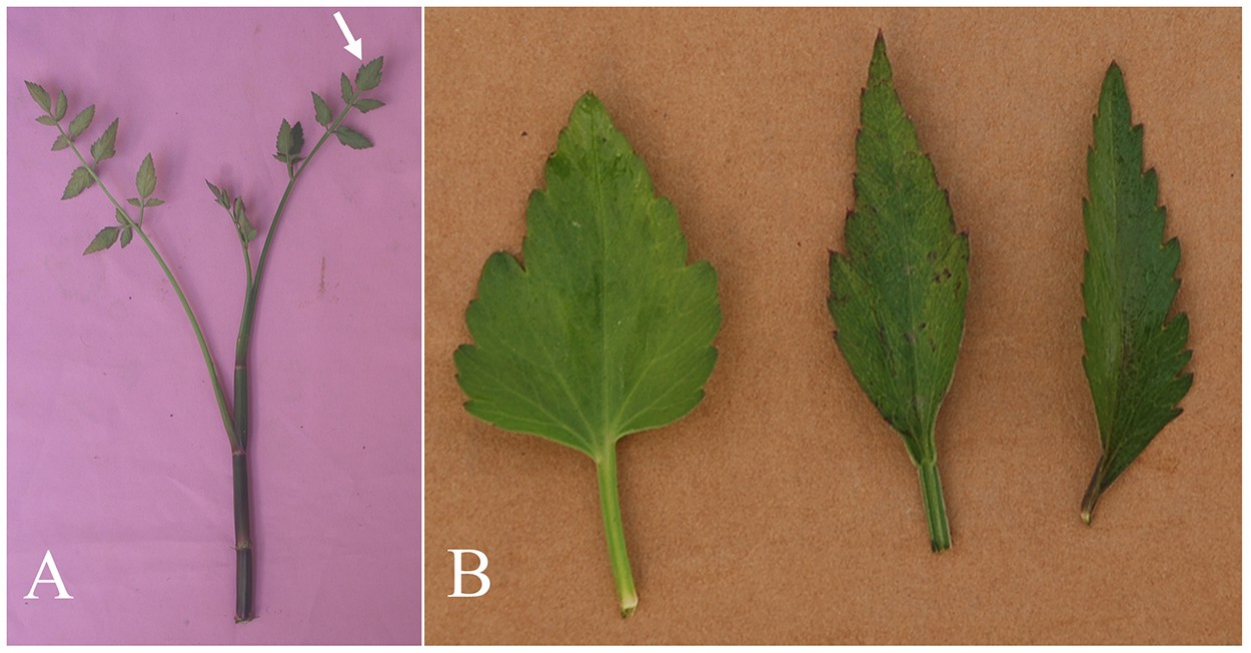
*Oenanthe javanica* phenotypes. (A) *O*. *javanica* accession, and ultimate segment shown with arrow. (B) From left to right: even ultimate segment with obtusely serrate margin, ultimate segment with shallowly serrate margin, and uneven ultimate segment with deeply serrate margin.

Our phenotypic trait investigation disclosed that leaf shape indicated by ultimate segment length:width ratio was more important than the separate ultimate segment length and width. Hence, the ultimate segment length:width ratio was used in the clustering analysis. The 108 *O*. *javanica* accessions were divided into two clusters, as this number was optimal and automatically selected by the TwoStep cluster analysis procedure in SPSS. The descending order of trait importance for cluster formation was leaf margin, leaf state, stem diameter, ultimate segment length:width ratio, flowering time, and plant height. Cluster 1 contained 73 accessions, while cluster 2 contained the remaining 35 (S2 Table). The accessions in cluster 1 produced even ultimate segments with obtusely serrate or shallowly serrate margins. The accessions in cluster 2 had mostly uneven ultimate segments with deeply serrate margins (Fig 3, Table 1).

**Table 1:** Summary statistics of *O*. *javanica* population phenotypic traits.

Trait	Cluster 1 (n = 73)	Cluster 2 (n = 35)
**Leaf margin**	Shallowly serrate (42.5%); obtusely serrate (57.5%)	Deeply serrate (100%)
**Leaf state**	Uneven (21.9%); even (78.1%)	Uneven (80%); even (20%)
**Ultimate segment length Mean (cm)**	2.71	2.85
**Ultimate segment width Mean (cm)**	1.88	1.52
**Ultimate segment length:width ratio Mean**	1.48	1.99
**Plant height Mean (cm)**	54.98	56.45
**Stem diameter Mean (cm)**	1.05	0.69

The mean ultimate segment lengths for clusters 1 and 2 were 2.71 cm and 2.85 cm, respectively, but they did not significantly differ (*P* = 0.282). The ultimate segment widths of the cluster 1 accessions were significantly larger than those of cluster 2 accessions. Their means were 1.88 cm and 1.52 cm, respectively (*P*<0.01). The mean of the ultimate segment length:width ratio for cluster 1 (1.48) was significantly smaller than that of cluster 2 (1.99) (*P*<0.01) (Table 1, S2 Table). Though ultimate segment lengths did not significantly differ between clusters, there was a significant difference between clusters in terms of ultimate segment width. Thus, the ultimate segments of cluster 1 were significantly wider than those of cluster 2.

The mean plant height of cluster 2 accessions (56.45 cm) was greater than that of the cluster 1 accessions (54.98 cm). Nevertheless, the differences between the clusters were not significant (*P* = 0.524). In contrast, the stem diameters significantly differ between the clusters (*P*<0.01) (cluster 1: 1.05cm, cluster 2: 0.69 cm) (Table 1, S2 Table). Therefore, *O*. *javanica* genotypes with long stems and wide stems are suitable for cultivation. Here, several accessions in both clusters were landraces.

Ye et al. [17] analyzed 98 *O*. *javanica* accessions and revealed that they could be classified as small-leaf or big-leaf types based on the leaf sizes and margins on ultimate segments. In the present study, it was difficult to categorized *O*. *javanica* accessions into subgroups based only on leaf sizes and margins, as these traits exhibited no clear morphological patterns. Therefore, the SPSS TwoStep cluster analysis procedure was implemented. Clusters 1 and 2 corresponded to big-leaf and small-leaf types, respectively. The accessions in cluster 1 and the big-leaf types had the same characteristics of leaf margins, stem diameters and ultimate segment widths. The accessions in cluster 2 and the small-leaf types also presented with the same leaf margins, stem diameters, and ultimate segment widths.

### RAD sequencing data and SNP characterization

We used *fastp* to remove low-quality reads from the raw data including adapter sequences, repetitive or ploy-N-containing reads, or empty reads [41], RAD sequencing then generated 446.03 million paired-end reads with each 150 bp in size. The ratio of sequencing nucleotides with quality value > Q30 was 89.30%. The GC content of the filtered reads from all accessions was in the range of 33.89–37.61%, and the average was 35.26%. The number of reads per accession was in the range of 1,701,586–7,057,194, and the average was 2,822,981. The read coverage per accession was in the range of 6.94–25.23×, and the mean sequencing depth was 12.81× (S3 Table). Of 123,310 SNPs, 11,493 were high quality and were detected based on the thresholds MAF≥0.05 and SNP integrity≥0.8 (S4 Table).

Water dropwort is mainly propagated asexually. For all accessions, 3.58% of the polymorphic SNP loci were heterozygous. On average, *O*. *javanica* had a lower frequency of heterozygous SNP loci (3.14%) than *O*. *linearis* (4.53%) (S5 Table). Their heterozygous SNP ratios were significantly lower than that of apple (22.35%). For *Oenanthe* spp., then, asexual reproduction decreased heterozygosity to a greater extent than hybridization [42].

### Germplasm phylogeny and population structure

**Fig 4:**
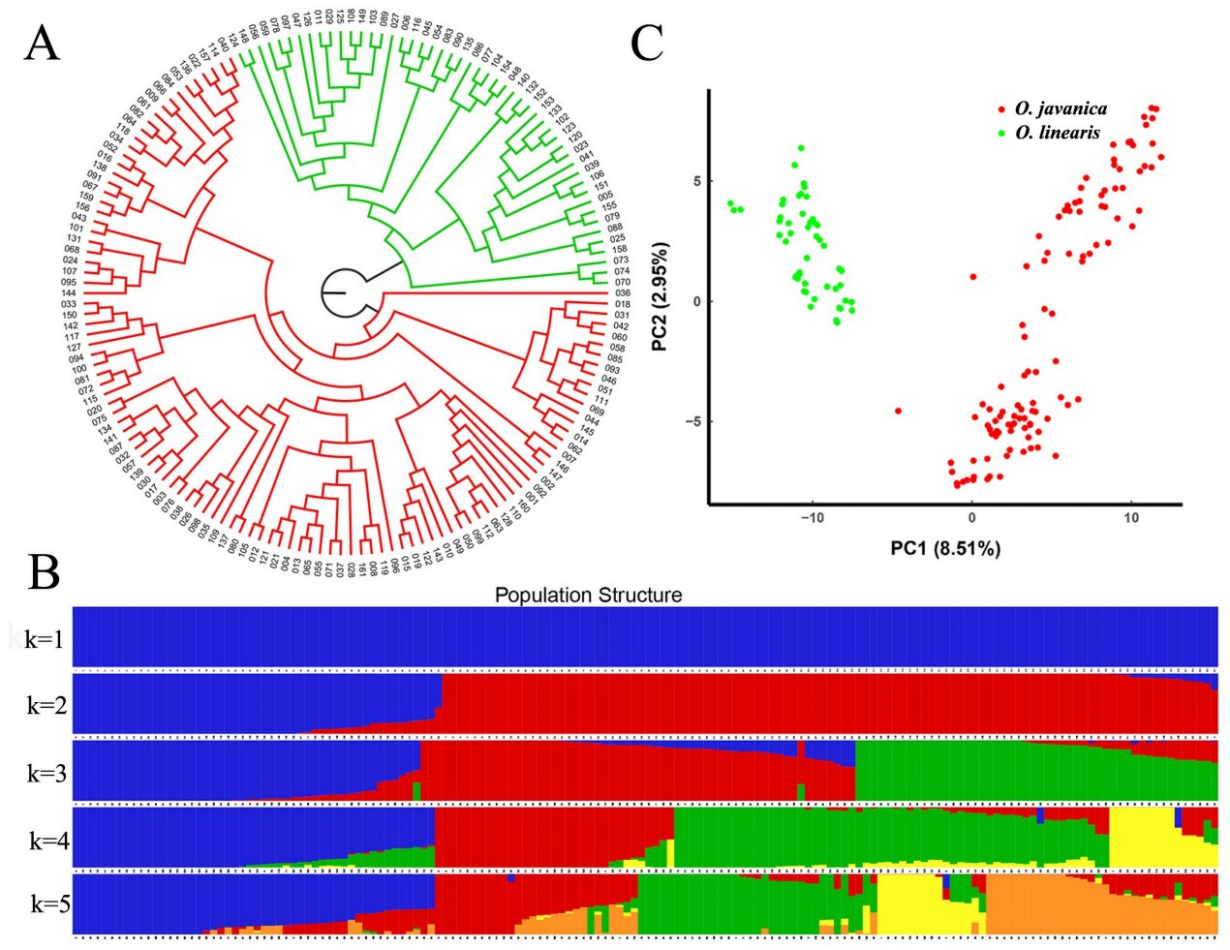
Population structure of 158 water dropwort accessions. (A) Phylogenetic tree of 158 water dropwort accessions inferred from 11,493 SNPs. *Oenanthe linearis* accessions are in green and *O*. *javanica* accessions are in red. (B) Population structure generated using ADMIXTURE. Each color represents one population. Individual accessions are represented by vertical bars. Ratio of each colored segment in each vertical bar represents the membership probability of accessions belonging to different populations. (C) PCA plots of first two components of 158 water dropwort accessions.

The phylogenetic relationships among the 158 accessions based on the 11,493 SNPs are shown in Fig 4A. Separation between species was mirrored in genotype-based clustering. *Oenanthe linearis* and *O*. *javanica* clustered into two independent clades. One clade comprised 50 *O*. *linearis* accessions from 12 provinces in China. The other clade consisted of 108 *O*. *javanica* accessions including 105 from 14 provinces in China, two from Vietnam, and one from Japan (S1 Table). The estimated membership fractions of the 158 accessions using the population structure demonstrated that the germplasm resources formed two populations (Fig 4B). Population Q1 contained 50 *O*. *linearis* accessions, whereas all 108 accessions in population Q2 were *O*. *javanica*. The population structure prediction was consistent with that of the phylogenetic tree analysis. Moreover, the PCA separated the 158 accessions into two populations as well. Thus, there was a high level of discrimination between species (Fig 4C).

The population structure models of the 158 accessions based on the 10 SNP subsamples were consistent with each other and with the result predicted according to the entire SNP set except accession 36. The latter was classified into one group based on population structure models predicted from two SNP subsamples and categorized into the other group according to the results for the other eight SNP subsamples (S6 Table). The genetic composition of accession 36 suggested the presence of a combination of two different genetic backgrounds accounting for 56% and 44% of the total (Fig 4B). The genetic compositions of several other accessions also indicated the occurrence of a mixture of various genetic backgrounds. Hence, gene flow must have taken place between *O*. *linearis* and *O*. *javanica* and influenced the genetic integrity of *Oenanthe* spp.

The phylogenetic and population structure analyses and PCA disclosed that all 158 accessions could be divided into *O*. *linearis* and *O*. *javanica*. The population structure models based on 10 different SNP subsamples validated this conclusion.

The complete *O*. *javanica* chloroplast genome sequence was compared with those of 29 Apiaceae genera (34 species). A phylogenetic analysis showed that *O*. *javanica* was nested in the tribe Oenantheae of Apiaceae and was closely related to *Cicuta virosa* and *Cryptotaenia japonica* [14]. Though the aforementioned study clarified *O*. *javanica* taxonomy in Apiaceae, it only used this species in *Oenanthe*.

A previous study [13] used nrDNA ITS sequences for 170 *Oenanthe* accessions (165 from 15 provinces of China, two from North Korea, one from Japan and two from Vietnam). The authors concluded that the accessions clustered into two groups closely related to *O*.*mildbraedii* and *O*.*sarmentosa* rather than *O*. *linearis* and *O*. *javanica*. Whereas the latter two are the most widely cultivated and used *Oenanthe* species in various regions of China, neither *O*.*mildbraedii* nor *O*.*sarmentosa* has been recorded in China. The aforementioned study comprised GenBank ITS sequences for nearly all currently reported *Oenanthe* species including one accession from *O*. *linearis* subsp. *linearis*, two from *O*. *linearis* subsp. *rivularis*, one from *O*.*sinensis*, one from *O*. *javanica*, one from *O*. *javanica* subsp. *javanica*, and one from *O*. *javanica* subsp. *stolonifera*. *Oenanthe linearis* subsp. *linearis* and *O*. *javanica* subsp. *javanica* were closely related, while the other five accessions were clustered together. This discovery is inconsistent with the logical assumption that accessions of the same species should be closely related. Plausible interpretations of this finding were that the number of markers used in that study was limited, or ≥ 1 of these seven accessions was misnamed. The ITS sequences of the seven accessions were processed by different researchers. Our results combined with those of the previous study suggest that further investigations over a wider geographic area are required to resolve the taxonomic discrepancies, as *Oenanthe* accessions are very widespread and have a very diverse morphology.

### *Oenanthe linearis* accession phylogeny and population structure

Different species predominated in the ADMIXTURE analysis. Therefore, the population structure models were analyzed either with *O*. *linearis* or *O*. *javanica* populations under the assumption of *K* in the range of 1–10. An optimal *K = 2* was proposed for *O*. *linearis* and indicated that this population could be categorized into two groups (S1 Fig). Group 1 contained 34 accessions and group 2 contained 16. Group 1 included 30 LL and 4 of 20 DCL. All 16 accessions in group 2 were DCL.

**Fig 5:**
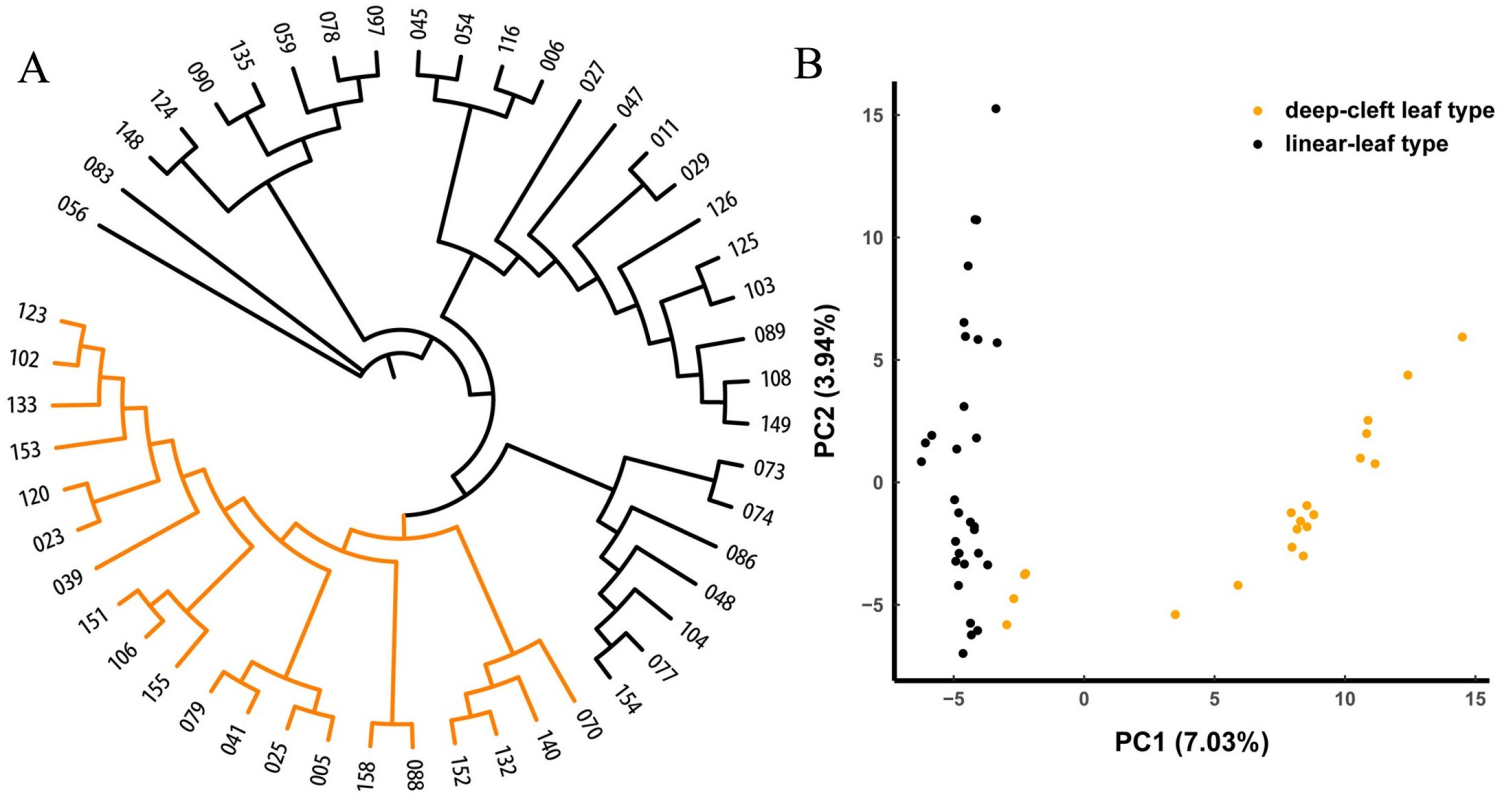
Population structure of 50 *O*. *linearis* accessions. (A) Phylogenetic tree of 50 *O*. *linearis* accessions. Group A (linear-leaf type) is shown in black and Group B (deep-cleft leaf type) is shown in orange. (B) PCA plots of first two components of 50 *O*. *linearis* accessions.

The *O*. *linearis* accessions were further classified into two groups with distinct geographic distribution patterns (Fig 5A, Table 2). Group A contained 30-LL accessions mainly from middle-southern and eastern China. Group B consisted of 20 DCL-type accessions mainly from southwestern and eastern China. Approximately, 27% of the LL and 40% of the DCL types originated from Jiangsu and Anhui Provinces in eastern China. Both of these regions are major water dropwort producers. Phylogenetic relationships based on genetic distances were relatively consistent with those predicted by the estimated population structure. The PCA suggested a two-cluster structure for *O*. *linearis* population (Fig 5B).

**Table 2:** Geographic origin of each accession in the four groups.

Group	Total	EC	MSC	SWC	NWC	NEC	Japan	Vietnam
**A**	30	8	16	5	--	1	--	--
**B**	20	8	3	9	--	--	--	--
**I**	61	23	19	18	1	--	--	--
**II**	47	24	8	10	2	--	1	2

EC, eastern China; MSC, middle-southern China; SWC, southwestern China; NWC, northwestern China; NEC, northeastern China.

Water dropwort with long, wide, and erect stems are suitable for cultivation. Therefore, wild plants with those morphological traits might have been locally collected and cultivated. Over time, they became landraces. Within *O*. *linearis*, four landraces intermingled with the wild accessions into the DCL-type group. Another three landraces were clustered into the LL-type group along with the wild accessions. Hence, the domestication period was short, and the degree of domestication was low for water dropwort.

### *Oenanthe javanica* accession phylogeny and population structure

The maximum likelihood of the estimated membership fractions of the 108 *O*. *javanica* was in the range of 1–10 for different *K* values. *K = 2* was optimal for this population (S2 Fig). Thus, *O*. *javanica* could be categorized into two groups, with 61 and 47 accessions.

**Fig 6:**
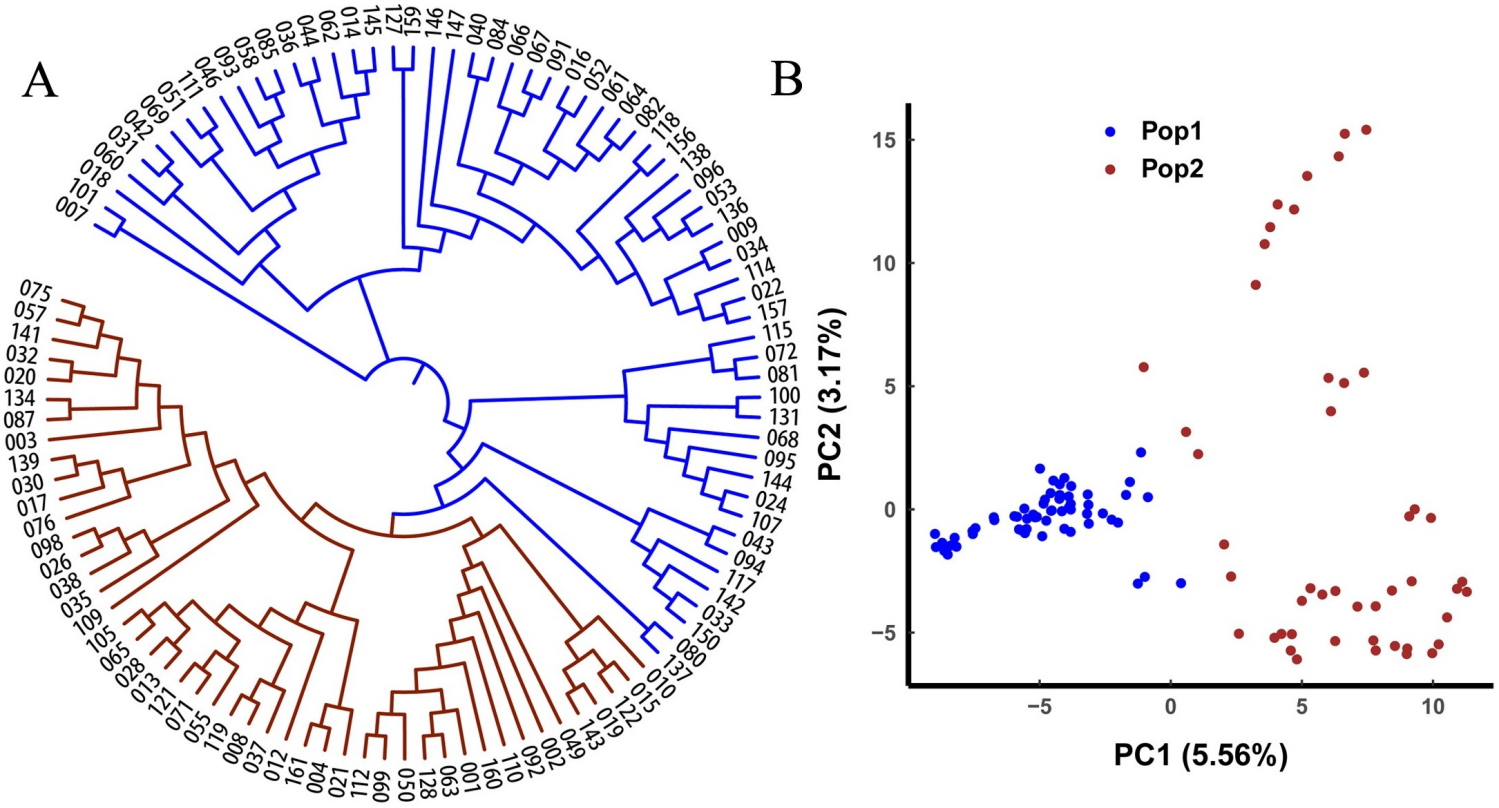
Population structure of 108 *O*. *javanica* accessions. (A) Phylogenetic analysis of 108 *O*. *javanica* accessions. Group I accessions are in blue, and group II accessions are in brown. (B) PCA plots of first two components of the 108 *O*. *javanica* accessions. Subpopulations defined by the phylogenetic tree include Pop1 (group I) and Pop2 (group II).

To clarify the phylogenetic relationship among *O*. *javanica* accessions, we used the NJ method in MEGA to construct a phylogenetic tree that could be divided into two groups (Fig 6A). The accessions in each group had distinct geographic distribution patterns (Table 2). Group I included 61 accessions mainly from eastern, middle-southern, and southwestern China, group II comprised 47 accessions mainly from eastern China. As Jiangsu and Anhui Provinces are the main water dropwort-producing areas, 36% of the *O*. *javanica* accessions were collected from there. Once again, landraces were clustered with the wild accessions into a single group. This finding was consistent with the assignments determined using ADMIXTURE. The PCA also separated the 108 *O*. *javanica* accessions into two major groups (Fig 6B).

The previous study was based exclusively on the morphological characters of the accessions and used a relatively small germplasm sample. It concluded that *O*. *linearis* could be classified into LL and DCL types, while *O*. *javanica* could be divided into small-leaf and big-leaf types [17]. To date, the population structure of the water dropwort germplasm has not been addressed in depth. In the present study, we first investigated the phenotypic traits of the water dropwort and then classified the germplasm resources. The two phenotypically based types of *O*. *linearis* substantially differed in terms of DNA sequence (Fig 5). For this reason, the morphological traits investigated here were suitable to classify *O*. *linearis*. For *O*. *javanica*, however, there was 28% discrepancy between the morphological classification and the SNP-based phylogenetic tree. Clusters 1 and 2 corresponded to groups II and I, respectively. Group I (61 accessions) included 33 accessions in cluster 2 (35 accessions) and 28 accessions in cluster 1 (73 accessions). Group II (47 accessions) comprised 45 accessions in cluster 1 (73 accessions) and 2 accessions in cluster 2 (35 accessions). An attribute importance analysis showed that leaf margin and state were the most important variables in cluster formation. However, these were categorical. Hence, the survey results could have been biased by the subjective judgments of the researchers. Greater objectivity could have been obtained by analyzing continuous variables. Overall, SNP-based classification was more accurate than morphological classification because phenotypic traits are affected by environmental conditions and subjective judgments. Joint morphological and SNP-based classification analyses suggested that other phenotypic traits of *O*. *javanica* must be investigated to identify the most important ones differentiating the subgroups. Those traits such as inflorescence number, flower number in inflorescence, seed setting rate, germination rate, and stolon number per plant should be considered in future research.

### Genetic diversity and population differentiation

Several genetic parameters were separately estimated to evaluate the genetic diversity and the extent of differentiation between the two species. The global genetic diversity indices (π) for the *O*. *linearis* and *O*. *javanica* populations were 0.1902 and 0.2174, respectively (Table 3). As both species could be further classified into different groups, we separately investigated the detailed relationship between the LL and DCL types and between groups I and II. Within *O*. *linearis*, the diversity of the LL type (π = 0.1818) was slightly lower than that of the DCL type (π = 0.1901). This discovery is consistent with Nei’s genetic diversity indices (0.18 for LL type and 0.20 for DCL type). Within *O*. *javanica*, the diversity of group I (π = 0.1985) was slightly lower than that of group II (π = 0.2318). This finding was also confirmed by Nei’s genetic diversity indices (0.27 for group I and 0.32 for group II). The Fst obtained from the comparison between LL and DCL types and between groups I and group II were 0.0347 and 0.0261, respectively. A relatively higher Fst (0.0471) was obtained from the comparison between *O*. *linearis* and *O*. *javanica*. A pairwise Nei’s genetic distance analysis between groups I and II indicated the lowest differentiation (0.0143). The Nei’s genetic distance was the highest between *O*. *linearis* and *O*. *javanica* (0.0303). DCL types had wider genetic diversity than LL types, while group II had wider genetic diversity than group I even when the numbers of DCL types and group II were smaller than those of LL types and group I. *Oenanthe linearis* and *O*. *javanica* had the highest differentiation, while groups I and II were the most closely related.

**Table 3:** Summary statistics for *O*. *linearis* and *O*. *javanica* populations based on SNP calculation.

Parameter	*O*. *linearis*			*O*. *javanica*		
	LL	DCL	Total	Group I	Group II	Total
**Nucleotide diversity (π)**	0.1818	0.1901	0.1902	0.1985	0.2318	0.2174
**Nei’s genetic diversity index**	0.18	0.20	0.20	0.27	0.32	0.30

LL, linear-leaf type; DCL, deep-cleft leaf type; groups I and II defined by phylogenetic tree.

The overall sequence diversity levels (π) for *O*. *linearis* and *O*. *javanica* were 0.1902 and 0.2174, respectively. In contrast, π = 0.1539 for apple [42], 0.0019 for soybean, and 0.0024 for rice [27]. The value of π varied only slightly among the four water dropwort groups (range 0.1818–0.2318). The π value for apple was low, as fruit quality underwent selection during domestication. The π values were extremely low for soybean and rice, as these two species encountered a major genetic bottleneck during domestication. This phenomenon may not have occurred in water dropwort. This plant is widely distributed in wild habitats and has been used as a vegetable for > 3,000 y. However, it has only been cultivated over the last ~300 y. Thus, water dropwort can now be harvested from both wild and cultivated sources as a vegetable crop.

## Conclusions

Phenotypic traits and SNP-based analyses were combined to investigate the phylogenetic relationships and genetic diversity of water dropwort. The SNPs generated by RAD-seq in the present study are the most abundant markers acquired for *O*. *linearis* and *O*. *javanica*. These species are most commonly distributed over a wide geographical area of China and its neighboring countries. SNP-based molecular characterization of the water dropwort collection examined here revealed broad sequence variations between *O*. *linearis* and *O*. *javanica*. This finding was consistent with phenotype diversification. The phylogenetic tree and the PCA plots disclosed that *O*. *linearis* is readily classified into linear-leaf and deep-cleft leaf types, and these traits are most effectively distinguished at reproductive growth. SNP-based analysis revealed that *O*. *javanica* is categorized into two groups that do not fully corresponded to the two clusters determined by the phenotypic trait clustering analysis. *Oenanthe javanica* displayed higher levels of genetic diversity (π = 0.2174) than *O*. *linearis* (π = 0.1902). However, there was a low level of population differentiation between these species (Fst = 0.0471). The present study provides novel insights into the phylogenetic relationships and genetic diversity of water dropwort.

## Supporting information

S1 Table 158 accessions of water dropwort germplasm set: Origins, groups, types, and species. (XLSX)

S2 Table Phenotypic traits of 158 accessions of water dropwort germplasm set. (XLSX)

S3 Table Summary statistics of RAD sequencing data. (XLSX)

S4 Table SNPs developed in the present study. (TXT)

S5 Table Summary SNP statistics. (XLS)

S6 Table Groups classified using population structure analyses based on 10 different SNP subsamples and total SNP set. (XLSX)

S1 Fig Population structure of 50 *O*. *linearis* accessions generated by ADMIXTURE. (TIF)

S2 Fig Population structure of 108 *O*. *javanica* accessions generated by ADMIXTURE. (TIF)
