# Supplementary figures and images for: Uncovering phylogenetic relationships and genetic diversity of water dropwort using phenotypic traits and SNP markers
Source: PLoS One. 2021 Jul 6;16(7):e0249825. doi: 10.1371/journal.pone.0249825 (PMC8259969; doi:10.1371/journal.pone.0249825)

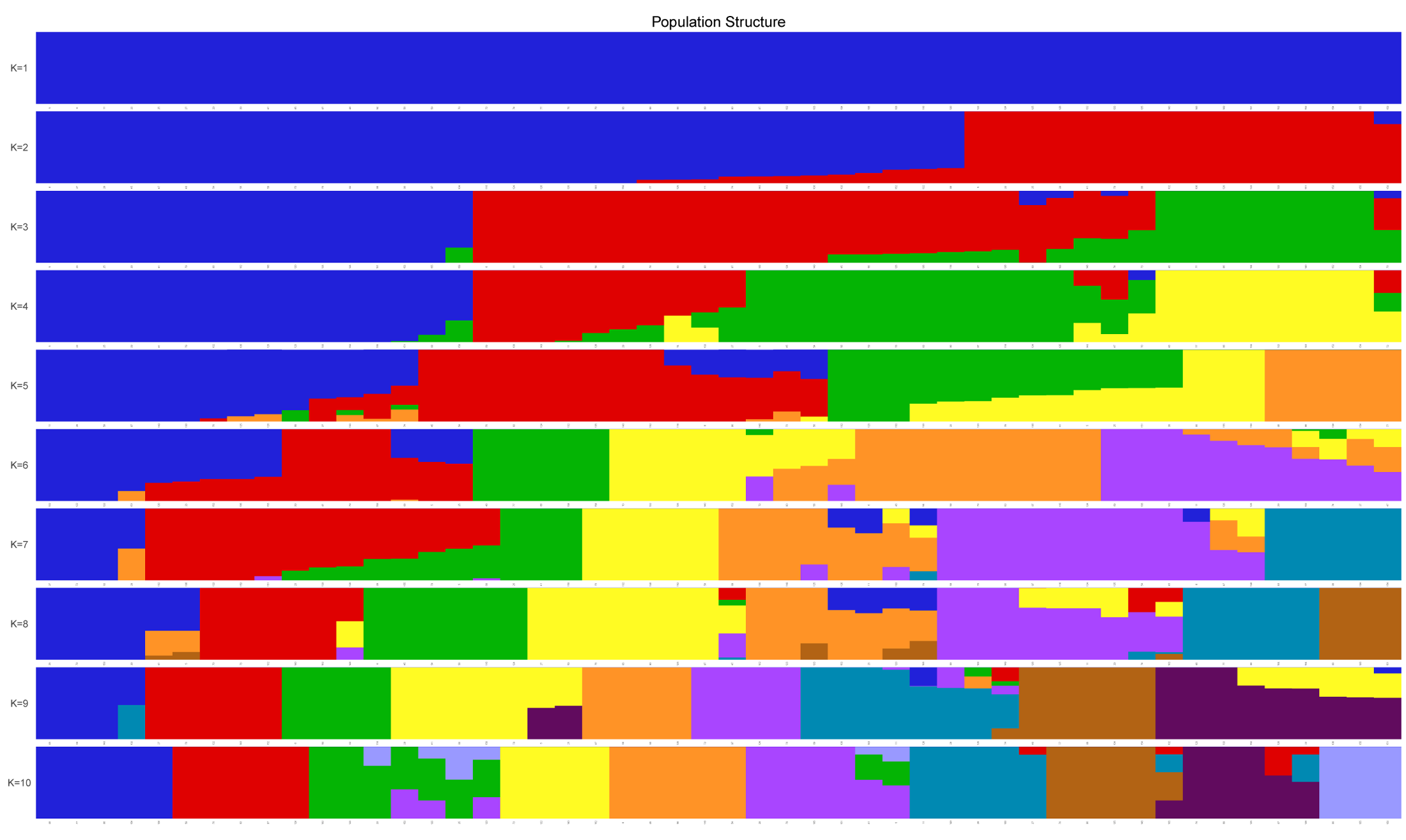

Supplement: S1 Fig — (TIF) [file pone.0249825.s007.tif]

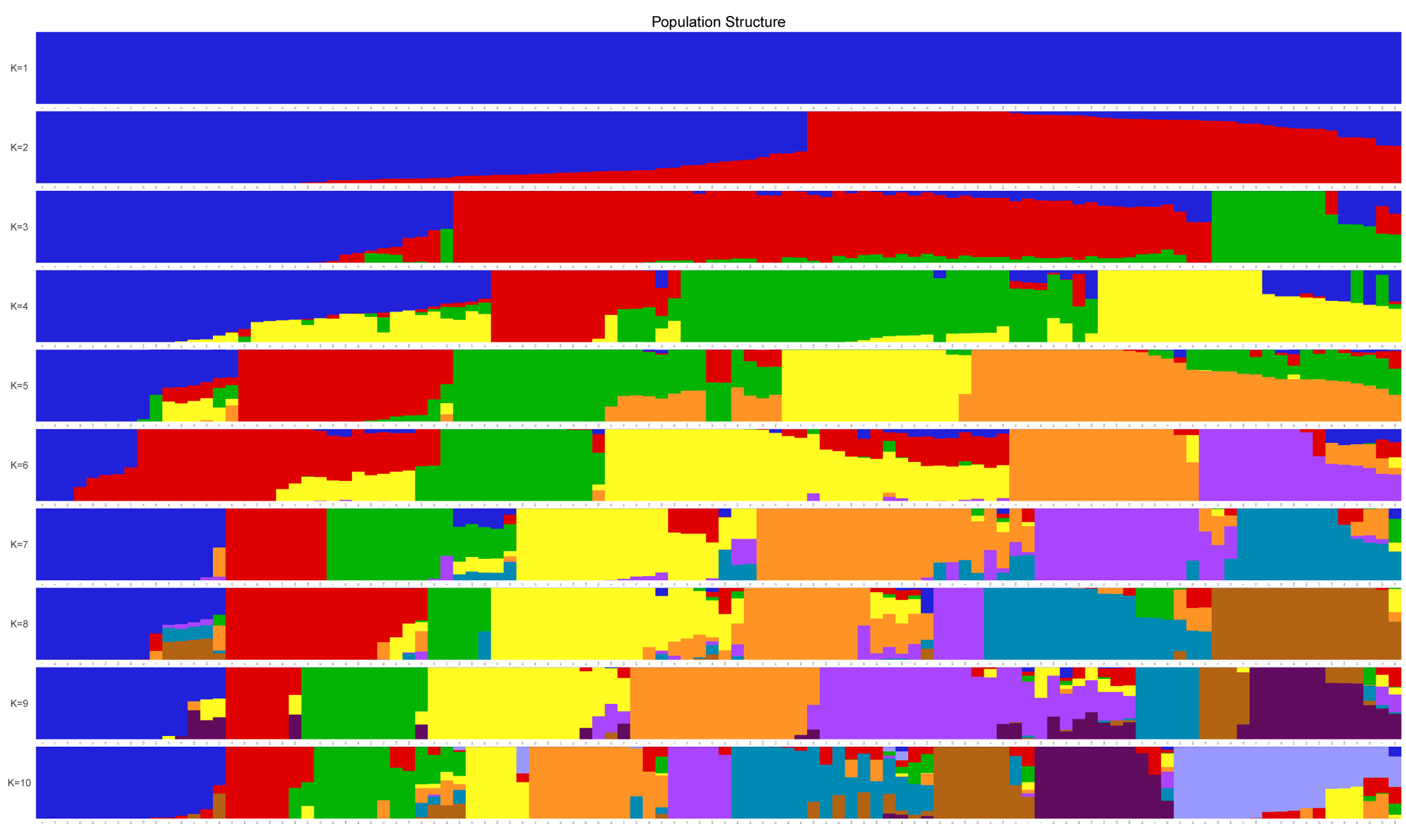

Supplement: S2 Fig — (TIF) [file pone.0249825.s008.tif]
